# Emotional distress and dysfunctional illness perception are associated with low mental and physical quality of life in Chinese breast cancer patients

**DOI:** 10.1186/s12955-017-0803-9

**Published:** 2017-12-01

**Authors:** Lili Tang, Kurt Fritzsche, Rainer Leonhart, Ying Pang, Jinjiang Li, Lili Song, Irmela Fischer, Maike Koch, Alexander Wuensch, Ricarda Mewes, Rainer Schaefert

**Affiliations:** 1 Psycho-Oncology Department, Key Laboratory of Carcinogenesis and Translational Research (Ministry of Education), Department of Psycho-Oncology, Peking University School of Oncology, Beijing Cancer Hospital & Institute, Beijing, China; 2 Center for Mental Health, Department of Psychosomatic Medicine and Psychotherapy, Medical Center - University of Freiburg, Faculty of Medicine, Freiburg, Germany; 3 Institute of Psychology, University of Freiburg, Freiburg, Germany; 4 Department of Psychology, University of Marburg, Marburg, Germany; 5 Department of Psychosomatic Medicine, Division of Internal Medicine, University Hospital Basel, Hebelstrasse 2, 4031 Basel, Switzerland

**Keywords:** Breast cancer, Oncology, Quality of life, Emotional distress, Illness perception

## Abstract

**Background:**

To evaluate the relationship between quality of life (QOL) and physical as well as psychological variables in Chinese breast cancer patients.

**Methods:**

This multicenter cross-sectional study enrolled 254 Chinese breast cancer patients in different stages and treatment phases. They answered standard instruments assessing QOL (EORTC), somatic symptom severity (PHQ-15), depression (PHQ-9), anxiety (GAD-7), health-related anxiety (WI-7), illness perception (BIPQ), and sense of coherence (SOC-9). Canonical correlation was applied to identify the strongest correlates between the physical, emotional and social QOL scales and the physical and psychological variables.

**Results:**

In our sample, a low global QOL was significantly associated with the following physical and psychological variables: symptom-related disability (Karnofsky Index) (*r* = .211, *p* < .01), somatic symptom severity (*r* = −.391, *p* < .001), depression (*r* = −.488, p < .001), anxiety (*r* = −.439, p < .001), health-related anxiety (*r* = −.398, p < .001), dysfunctional illness perception (*r* = −.411, p < .001), and sense of coherence (*r* = .371, p < .001). In the canonical correlation analysis, high somatic symptom severity, depression, anxiety, dysfunctional illness perception, and low sense of coherence showed the strongest correlations with low physical, emotional and social functioning. The first three significant canonical correlations between these two sets of variables were .78, .56, and .45.

**Conclusions:**

QOL in Chinese breast cancer patients is strongly associated with psychological factors. Our results suggest that Chinese physicians and nurses should incorporate these factors into their care for women with breast cancer to improve patients’ QOL.

## Background

China has traditionally been believed to have a low incidence of breast cancer [1]. However, in the past two decades, the age-standardized incidence of breast cancer has increased five-fold, from 6.4 per 100,000 in 1980 to 31.93 per 100,000 in 2011 [2, 3]. The reasons for this increase are multi-factorial and include longer exogenous as well as endogenous estrogen exposure due to “westernized lifestyles”, the one-child policy, better provision and uptake of screening for breast cancer, better awareness about breast cancer in the Chinese population, and better diagnostic procedures [4, 5]. Considering the large population base of 1.34 billion, China is facing an increasing and enormous social and economic burden of breast cancer.

Given this background, improving the quality of life (QOL) of breast cancer survivors has significant social and public health implications. QOL is a subjective assessment of physical, psychological, and social well-being [6] and reflects patients’ perceptions of the impact of breast cancer diagnosis and treatment on daily living [7, 8]. In Western countries, there are clear connections between QOL, depression, anxiety and somatic symptoms such as pain and fatigue in breast cancer patients [9–12]. In a study on the 4-week prevalence of mental disorders in cancer populations, the highest prevalence of any mental disorder was found in patients with breast cancer (41.6%; 95% CI, 36.8 to 46.4%) [13]. Similar to patients from Western countries, breast cancer survivors in China experience a high level of depressive and anxiety symptoms [14]. Higher levels of depression and anxiety have been positively associated with a higher level of passive coping style and negatively associated with perceived social support [15].

Several breast cancer studies in Western countries as well as in China have reported that some sociodemographic variables are associated with QOL such as age at cancer diagnosis, level of education, income, employment status, marital status, and social support [9, 11, 16].

In addition, psychological factors such as illness perception [17] and sense of coherence have been found to be strongly associated with QOL in cancer patients [18, 19]. Illness perception is defined as an individual’s cognitive (i.e., beliefs, ideas, thoughts) and emotional (i.e., feelings) perceptions of an illness [17]. Illness perceptions in women with breast cancer have been shown to be an important co-variable of QOL [20]. Higher scores on negative and threatening illness perceptions have been associated with lower QOL [21]. In cancer patients, health-related anxiety may be accompanied by inaccurate body perceptions, and patients’ perceptions of symptoms combined with their concerns about illness may lead them to interpret harmless body sensations as symptoms of cancer progression [22]. While empirical studies of predominantly Anglophone cancer patients have indicated that negative illness perceptions are significantly associated with more psychological distress and worse QOL [23, 24], it remains unclear how non-Western breast cancer patients interpret their symptoms.

According to the “salutogenic model”, sense of coherence (SOC) plays an important role in human health [18, 25]. Antonovsky defined SOC as "a global orientation that expresses the extent to which one has a pervasive, enduring though dynamic feeling of confidence that (1) the stimuli deriving from one's internal and external environments in the course of living are structured, predictable and explicable; (2) the resources are available to one to meet the demands posed by these stimuli; and (3) these demands are challenges, worthy of investment and engagement" [25]. In a systematic review, SOC was strongly related to perceived health, particularly with regard to mental health [19].

To date, there have been no studies about QOL in Chinese breast cancer patients that focus on the relationship between QOL and physical as well as psychological variables.

### Study objectives

The overall objective of this study was to evaluate the associations between QOL and physical as well as psychological characteristics of Chinese breast cancer patients.

Our specific research aims were as follows:

1. To analyze the correlations in metric variables and mean differences in categorical variables between QOL and sociodemographic and clinical data, somatic symptom severity, depression, anxiety, health-related anxiety, illness perception, and SOC in Chinese breast cancer patients; and
2. To evaluate which of these physical and psychological variables have the strongest correlations with QOL, as measured by the global score and the functional and symptom scales of the European Organization for Research and Treatment of Cancer (EORTC) Core QOL Questionnaire.

## Methods

### Study design and setting

This study is part of a Sino-German research network entitled “Patients with multiple somatic symptoms in China” [26–28]. We performed a multicenter, cross-sectional study in three Beijing hospitals (Beijing Cancer Hospital: Department of Psycho-oncology and Department of Traditional Chinese Medicine [TCM]); People’s Liberation Army (PLA) Military Hospital: Departments of Medical Oncology and Gynecology; Beijing Tiantan Hospital: Breast Surgery Department). The study was approved by the ethic committees of the universities affiliated with the two principal investigators (LT and KF), the Beijing Cancer Hospital and the University Medical Centre, Freiburg, Germany.

### Subjects

The study was performed under routine clinical conditions on randomly assigned screening days between July 1, 2012, and June 30, 2013. Patients who entered a participating department of the abovementioned hospitals on one of the screening days were informed about the study by research assistants and were asked to participate. Written informed consent was obtained from all participants. Research assistants were well-trained clinical research nurses or medical master’s students who were under the supervision of LT.

The inclusion criteria were age older than 18 years, a diagnosis of breast cancer without any stage criteria, and adequate reading and writing skills. The exclusion criteria comprised severe physical and cognitive impairment, psychosis and acute suicidal tendencies.

*.*

### Sociodemographic data and physical characteristics

The medical records were used to determine Karnofsky Performance Index Score (ranging from 0 [“death”] to 100 [“perfect health”]), cancer stage [0 to 4 or unknown], tumor size (Tis, T1-T4), lymph node status (NX, N0-N3), metastases (MX, M0, M1) (for more information, see http://www.uicc.org), treatment (surgery, hormonal therapy, chemotherapy, radiotherapy), and other severe diseases.

### Assessment instruments

We used the following questionnaires:

#### The European Organization for Research and Treatment of cancer (EORTC) Core quality of life questionnaire (QLQ-C30)

The EORTC QLQ-C30 (version 3.0) is a 30-item questionnaire composed of multi-item scales and single items that reflect the multidimensionality of the QOL construct. It includes five functional scales (physical, role, cognitive, emotional, and social), three symptom scales (fatigue, pain, and nausea/vomiting), and a global health status/QOL scale. The remaining single items assess additional symptoms commonly reported by cancer patients (dyspnea, appetite loss, sleep disturbance, constipation, and diarrhea), as well as the perceived financial impact of the disease [29]. A higher score on the functional subscales and the global QOL represent a higher level of functioning and QOL. A higher score on a symptom subscale or item represents a higher (more severe) symptom level [30]. The standard Chinese version of the EORTC QLQ-C30 (version 3.0) is a valid instrument used to assess QOL in Chinese breast, ovarian, lung, head and neck, colorectal, stomach and brain cancer patients [31-33]; there is no Chinese disease-specific module for breast cancer.

#### Somatic symptom severity scale of the patient health questionnaire (PHQ-15)

Patients were screened using the somatic symptom scale of the Patient Health Questionnaire (PHQ-15) [34]. The PHQ–15 is regarded as a somatization scale that was developed to assess the somatic symptoms that frequently accompany mental disorders rather than serious medical diseases. Two Chinese studies have shown that the Chinese version of the PHQ-15 exhibits satisfactory reliability and have provided preliminary evidence of its validity in the general population and in general health care [35, 36].

#### Depression scale of the patient health questionnaire (PHQ-9)

This instrument assesses each of the nine DSM-IV depression criteria [37]. The PHQ-9 has been shown to be a reliable and valid instrument in Chinese patients [38].

#### Anxiety scale (GAD-7)

The seven-item anxiety scale (GAD-7) was used to assess the severity of generalized anxiety and other common anxiety disorders [39]. The instrument has shown good reliability and criterion, construct, factorial, and procedural validity in a Chinese general hospital population [40].

#### Health-related anxiety (WI-7)

Health-related anxiety was assessed with the 7-item Whiteley-7 index [41]. The Chinese version of the WI-7 has exhibited satisfactory reliability and internal validity in the general population [42].

#### The brief illness perception questionnaire (BIPQ)

The brief illness perception questionnaire (BIPQ) [17] is an 8-item measure designed to provide a rapid assessment of a patient’s cognitive and emotional representations of illness. High scores represent a more threatening and negative illness perception. The Chinese BIPQ has shown acceptable reliability and validity [43].

#### Sense of coherence (SOC-9)

Sense of coherence was measured using a short version of the Sense of Coherence Scale (SOC-9) [44]. The SOC-9 provides a single-factor solution, with high scores reflecting a strong SOC. Cronbach’s α for the Chinese version was 0.82 [45].

##### Sample size calculation

The estimated effect for the sample size calculation was based on our previous study about somatic symptom severity in general hospital outpatients in China [26]. For the correlation analysis approach in this article a sample size of 211 achieves 90% power to detect a correlation of .20 using a one-sided hypothesis test with a significance level of .05. For the canonical correlation analysis at least 10 observations per independent variable are helpful to avoid “overfitting” the data [46]. For our canonical correlation analysis with 16 independent variables and our sample of 250 we can come out to 15 participants per independent variable. This is enough for a canonical correlation analysis [46].

### Statistical analyses

Statistical analyses were performed with IBM SPSS Statistics 23.0 and Stata. Categorical variables were evaluated using the χ^2^-test. Continuous variables were evaluated using analysis of variance. Correlation coefficients were calculated to assess the relationships between QOL, clinical characteristics and psychological variables.

The interrelations between the different scales of the EORTC and the physical as well as the psychological variables were analyzed by canonical correlation. This type of analysis estimates the correlations between two sets of variables to determine what is common among these two sets and to test the parameters for significance. *Canonical correlations help us to find relations between two sets of variables and to answer the question how many dimensions these relations have.* Canonical correlation analysis produces model equations between the two sets of variables with the aim of a maximum correlation between both linear combinations. The stepwise method starts with a first pair of canonical variables and tests the canonical correlation for significance. After this first step, a second pair of canonical correlations is estimated with the constraint that they are not correlated with the first pair of canonical variables. The procedure stops at the minimum number of variables in both sets. All canonical correlations are tested for significance, and only significant correlations should be interpreted. For the canonical correlation analysis, we used the questionnaire scales only and not single items because the measurement error of single items seemed to be too high. Statistical analyses were conducted using an alpha level of 1% to avoid alpha inflation resulting from multiple testing. For the canonical correlation, we used an alpha level of 5%.

## Results

### Study sample

A total of 269 breast cancer patients met the inclusion criteria, and 254 patients (94.8%) provided informed consent. The documentation of cancer stage in the charts was incomplete (22% unknown), but the patients with missing data and the ones with complete data did not differ in QOL, sociodemographic, clinical or psychological variables. For the other variables, the percentages of missing data were less than 3%, with the exception of the Karnofsky index (7.5% missing).

### Sociodemographic characteristics

The relationships between sociodemographic characteristics and the EORTC global score are shown in Table 1. According to the 1% alpha level, only employment status showed a significant correlation with QOL. Retired and unemployed women had a better QOL.

**Table 1:** Relationship between sociodemographic characteristics and quality of life (EORTC) global score

Variable	Total *N* = 254	EORTC global score Pearson correlation	t-value (df)	*p*-value
Age				
Mean, years (SD)	49.84 (10.10)	.105	1.66 (247)	.098
Variable	Total N (%)	EORTC global score Mean (SD)	F-value (df)	p-value
Health insurance				
Yes	215 (86.7%)	56.61 (25.61)	0.075	.785
No	33 (13.3%)	55.30 (25.67)	(1.244)	
Residence				
Urban	213 (85.9%)	56.57 (25.21)	0.035	.852
Rural	35 (14.1%)	55.71 (25.23)	(1.246)	
Marital status				
Single	8 (3.2%)	60.42 (25.49)	1.283	.277
Married	229 (90.9%)	56.15 (25.28)	(5.247)	
Married but separated	3 (1.2%)	77.78 (20.97)		
Divorced	7 (2.8%)	44.05 (25.78)		
Widowed	5 (2.0%)	68.33 (29.11)		
Life situation				
Alone	13 (5.2%)	51.92 (27.04)	2.774^b^	.028
With partner	72 (28.6%)	61.23 (22.95)	(4.245)	
Alone with children	15 (6.0%)	70.56 (27.61)		
With partner and children	134 (53.2%)	53.67 (24.85)		
With parents	16 (6.3%)	48.96 (28.03)		
Other	2 (0.8%)	54.17 (64.82)		
Family Income (monthly)				
Low (under 4000 RMB^a)^	131 (53.0%)	55.41 (26.67)	3.804	.024
Middle (4000–8000 RMB)	80 (32.4%)	62.19 (23.83)	(2.244)	
High (above 8000 RMB)	36 (14.6%)	48.84 (22.81)		
Employment				
Employed	87 (35.4%)	47.99 (26.53)	5.600^b^	.001*
Unemployed	31 (12.6%)	61.02 (20.79)	(3.240)	
Retired	113 (45.9%)	61.80 (24.13)		
Homemaker	13 (5.3%)	58.33 (22.82)		
Student	1 (0.4%)	83.33 (−)		
Other	1 (0.4%)	0.00 (−)		
Education				
Elementary school	14 (5.7%)	51.79 (25.36)	2.284	.080
Middle school	53 (21.5%)	59.75 (27.09)	(3.243)	
High school	89 (36.0%)	60.49 (24.41)		
University degree	91 (36.4%)	51.74 (24.74)		

All % values are column percentages. *p < .01

^a^RMB: The renminbi is the currency of the People’s Republic of China; 1000 RMB is equivalent to approximately 125 Euro

^b^Cells with two or less participants were excluded from the analysis of variance

### EORTC descriptive statistics

The values of all scales and items and their correlations with the global score are shown in Table 2. The global score showed moderate to high correlations with most of the scales and items. Only 2 items showed no correlation (diarrhea) or low correlation (financial difficulties) with the global score.

**Table 2:** EORTC descriptive statistics

	Effective N	M (SD)	Pearson correlation with EORTC global score
EORTC			
Global Score	253	56.32 (25.42)	
Role Functioning Score	247	68.92 (31.44)	.424***
Social Functioning Score	251	60.05 (31.71)	.521***
Cognitive Functioning Score	244	73.37 (22.02)	.391***
Emotional Functioning Score	251	74.66 (21.71)	.443***
Physical Functioning Score	252	72.82 (21.62)	.411***
Fatigue Score	242	40.50 (24.58)	−.614***
Pain Score	245	29.86 (25.08)	−.478***
Nausea & Vomiting Score	252	16.07 (24.28)	−.425***
Item dyspnea	250	20.00 (23.14)	−.307***
Item insomnia	248	33.60 (30.88)	−.266***
Item appetite loss	252	26.72 (31.77)	−.468***
Item constipation	254	24.02 (30.16)	−.325***
Item diarrhea	251	11.02 (21.25)	−.107
Item financial difficulties	254	48.16 (39.25)	−.199**

*M* mean, *SD* standard deviation

***p < .001, **p < .01

### Clinical data and the EORTC global score

More than half of the patients (56.5%) were diagnosed with stage 1 or 2 cancer. The vast majority of the sample received some type of surgical procedure (*n* = 227, 89%) as well as chemotherapy or hormone therapy (*n* = 226, 89%); 102 (40%) patients received radiotherapy.

QOL was not significantly associated with the following oncological characteristics: cancer stage, cancer size, involved lymph nodes, metastases, radiotherapy, surgery and other severe diseases. Patients who received chemo- and/or hormone therapy had a trend to worse QOL than patients who did not. However, QOL was associated with Karnofsky score (See Tables 3 and 4).

**Table 3:** Stage of cancer, treatment and EORTC global score

	N (%)	M (SD)	F (df1, df2)	*p*
Cancer stage				
Stage 0	2 (0.8%)	83.33 (23.57)	1.287 (5.247)	.270
Stage 1	38 (15.0%)	57.24 (26.44)		
Stage 2	105 (41.5%)	56.35 (25.59)		
Stage 3	28 (11.1%)	53.27 (22.71)		
Stage 4	24 (9.5%)	47.92 (26.72)		
Stage unknown	56 (22.1%)	59.82 (24.77)		
Chemo				
No	27 (10.7%)	66.67 (28.50)	5.086 (1.251)	.025
Yes	226 (89.3%)	55.09 (24.81)		
Radiology				
No	151 (59.7%)	57.73 (25.82)	1.140 (1.251)	.287
Yes	102 (40.3%)	54.25 (24.79)		
Surgery				
No	26 (10.3%)	56.09 (24.22)	0,002 (1.2651)	.961
Yes	227 (89.7%)	56.35 (25.42)		

*M* mean, *SD* standard deviation

**Table 4:** Relationship between psychological variables, Karnofsky Performance Index Score and EORTC global score

	Effective N	Mean (SD)	Pearson correlation with EORTC global score
Depression (PHQ-9)	252	7.39 (5.80)	−.488***
Anxiety (GAD-7)	252	4.87 (4.77)	−.439***
Health-related anxiety (WI-7)	253	4.29 (2.07)	−.398***
Brief IPQ (total score)	251	37.00 (12.46)	−.411***
PHQ-15	253	8.40 (4.97)	−.391***
Sense of coherence (SOC)	253	45.55 (9.73)	.371 ***
Karnofsky index	234	90.95 (7.55)	.211**

*M* mean, *SD* standard deviation

***p < .001, **p < .01

Low QOL (EORTC global score) in breast cancer patients was significantly associated with all analyzed psychological variables including high somatic symptom severity (PHQ-15) as a measure of somatization, depression (PHQ-9), anxiety (GAD-7), health-related anxiety (WI-7), threatening and negative illness perceptions (IPQ), and lower SOC (see Table 4).

### Canonical correlation

In the canonical correlation analysis of all significant variables, the strongest associations were found between the EORTC functioning (physical, emotional and social functioning) and somatic scales (fatigue, pain and nausea/vomiting) and the psychological variables such as somatic symptom severity, emotional distress (depression and anxiety), illness perception, and SOC (see Table 5).

**Table 5:** Canonical correlation between EORTC and other questionnaires

Variable EORTC	Coefficient (SE)	t-value (p)	Other variable	Coefficient (SE)	t-value (p)
Dimension 1 CC = .7802					
Global Score	−0.005 (0.003)	−1.65 (.101)	**PHQ-15**	**0.085 (0.013)**	**6.77 (<.001)**
Physical Functioning	−0.004 (0.004)	−1.13 (.260)	PHQ-9	0.009 (0.013)	0.70 (.486)
Role Functioning	−0.003 (0.003)	−1.08 (.281)	GAD-7	−0.010 (0.013)	−0.73 (.466)
**Emotional Functioning**	**−0.016 (0.003)**	**−4.88 (<.001)**	WI-7	0.040 (.035)	1.15 (.250)
Cognitive Functioning	−0.002 (0.003)	−0.61 (.543)	**BIPQ**	**0.036 (0.006)**	**6.41 (<.001)**
**Social Functioning**	**−0.008 (0.002)**	**−3.23 (.001)**	**SOC-9**	**−0.035 (0.007)**	**−5.23 (<.001)**
Fatigue	0.006(0.004)	1.49 (.138)	Karnofsky	−0.000 (0.000)	−1.15 (.251)
Pain	0.004 (0.003)	1.39 (.165)			
**Nausea/vomiting**	**0.008 (0.003)**	**2.68 (.008)**			
Dimension 2 CC = .5627					
Global Score	0.009 (0.006)	1.60 (.110)	**PHQ-15**	**0.065 (0.023)**	**2.81 (.005)**
Physical Functioning	−0.006 (0.007)	−0.89 (.373)	**PHQ-9**	**0.186 (0.024)**	**7.74 (<.001)**
Role Functioning	0.009 (0.005)	1.95 (.052)	**GAD-7**	**−0.185 (0.024)**	**−7.67 (<.001)**
**Emotional Functioning**	**0.046 (0.006)**	**7.51 (<.001)**	WI-7	−0.006 (.063)	−0.10 (.919)
Cognitive Functioning	−0.010 (0.006)	−1.58 (.115)	BIPQ	−0.011 (0.010)	−1.10 (.274)
Social Functioning	−0.006 (0.004)	−1.32 (.188)	**SOC-9**	**0.041 (0.012)**	**3.36 (.001)**
**Fatigue**	**0.029 (0.007)**	**4.14 (<.001)**	Karnofsky	−0.000 (0.000)	1.08 (.281)
Pain	0.006 (0.005)	1.15 (.250)			
**Nausea/vomiting**	0.008 (0.005)	1.45 (.149)			
Dimension 3 CC = .4476					
Global Score	−0.003 (0.008)	−0.39 (.699)	**PHQ-15**	**0.148 (0.031)**	**4.70 (<.001)**
**Physical Functioning**	**0.025 (0.010)**	**2.60 (.010)**	**PHQ-9**	**−0.100 (0.033)**	**−3.07 (.002)**
Role Functioning	0.007 (0.006)	1.14 (.256)	**GAD-7**	**0.103 (0.033)**	**3.13 (.002)**
Emotional Functioning	−0.003 (0.008)	−0.34 (.735)	WI-7	0.167 (.086)	1.94 (.054)
Cognitive Functioning	−0.009 (0.008)	−1.06 (.291)	**BIPQ**	**−0.067 (0.014)**	**−4.82 (<.001)**
**Social Functioning**	**0.026 (0.006)**	**4.44 (<.001)**	SOC-9	−0.014 (0.016)	−0.83 (.406)
Fatigue	0.003 (0.010)	0.26 (.795)	Karnofsky	0.000 (0.001)	0.51 (.608)
**Pain**	**0.029 (0.007)**	**4.06 (<.001)**			
**Nausea/vomiting**	**0.022 (0.007)**	**3.11 (.002)**			

*CC* canonical correlation; significant parameters are presented in bold

Dimensions 1–3: These three dimensions describe the three significant linear combinations between both sets of variables. Two vectors (sets) of variables and the correlations between these variables were described using linear combinations of the variables. The correlations were maximized and described in independent canonical correlations, presented here in three dimensions

## Discussion

We investigated the associations between QOL and physical as well as psychological variables in Chinese breast cancer patients.

The majority of patients in the study were married, well educated, and diagnosed with an early stage breast cancer. The study population was representative of Chinese breast cancer patients regarding sociodemographic data such as age, cancer stage and treatment. The mean age in our study was 49.8 years. In five epidemiological studies and reviews the mean age of Chinese breast cancer patients was about 50 years [4, 5, 47–49].

More than half of the patients in our study were in the earlier stages of cancer (56.5%). This percentage lies under the percentage of two large epidemiological studies [49, 50] with 61.5% and 80.1% stage 1 and 2 cancer. *Even if it consistent with other studies, findings relate primarily to early stages of cancer.*

Also the treatment distribution was similar to the large epidemiological study (*n* = 2252) of Peng et al. 2016 [49].

### Correlations of low QOL

In terms of our first research question, low QOL in Chinese breast cancer patients was found to be associated with higher somatic symptom severity and a lower Karnofsky performance index, with higher emotional distress, increased health-related anxiety, more negative and threatening illness perceptions, and a lower SOC. No significant associations were found between sociodemographic characteristics and QOL, with the exception of employment status.

*Within this sample size of about 250 and some very small subgroups in the variable “life situation” controlling for non-metric potential confounders such as living arrangements is complicated. We should estimate a canonical correlation within each group. This is not possible with this sample size. Within a replication with a higher sample size this question can be addressed. It seems that being alone with children would be associated with higher QOL. This is an unexpected result. But the variance in this sample is high. Two of them have really low values. Because we have only 15 participations in this group we think we should not over-interpret this result.*

Unemployed and retired patients surprisingly had a better QOL than employed persons. One reason for this finding could be that during the economic reforms work-related cognitive symptoms existed in breast cancer survivors, which made it difficult for them to adapt their work [51, 52]. But also during the economic reforms many women between 40 and 60 years lost their jobs and retired earlier. They cared for their grandchildren and had unregistered, temporary jobs to earn some money. Consequently, they had more leisure time, no job stress, fewer daily hassles and emotional and physical wellbeing increase. No data were found to verify this hypothesis.

### QOL and biomedical data

The results in the Chinese literature about the associations between QOL and cancer stage are inconsistent. In the study by Huang et al. 2013 [53] the QOL in the psychological, social and physical domains of patients at stage 0 and 1 were higher than patients at stage 3 and 4. In the study by Yan et al. 2015 [54] no significant differences were found for QOL in the four cancer stages.

*We belief that in a sample of about 250 participants an effect should be significant if this effect is existing in the population. The effects we found in our study are very low with a partial eta-square of .025. For this effect size and our sample size of 250 the analysis had only a power of .34. So for this effect size we would need a sample of nearly 1000 participants to reach a power of .9. A replication study can answer the question, if the not significant associations of QoL with cancer stage or size is related to a high proportion of earlier stage cancers, or could reflect a lack of statistical power in the sample.*

Chemotherapy was not significantly associated with QOL. But the group of patients without chemo- and/or hormone therapy was small. A study among Chinese breast cancer patients during adjuvant therapy found that the percentage of participants with anxiety or depression was significantly higher in the chemotherapy group than in the other treatment groups [55].

*The high percentage with a stage being unknown (22%) was a problem for the analysis. We did some analysis with and without this group of participants but there are no real differences in the results. Because we want do the analysis for the primary research questions with higher statistical power we include these participants in the analysis.* Post hoc *tests (Tukey) show that there are no pairwise group differences between the stage groups. All pairwise differences tests for the EORTC global have p > = .4 as result.*

### QOL and psychological variables

Regarding our second research question, canonical correlation analysis found that all psychological variables including somatic symptom severity were strongly correlated with the physical, emotional and social functioning scales of the EORTC. Multiple somatic symptoms have been shown to be correlated with physical QOL after adjusting for anxiety, depression and disease severity [56]. In our previous studies, we investigated a mixed sample of Chinese General Hospital outpatients and found that high somatic symptom severity was associated with adverse psycho-behavioral characteristics and low physical and mental QOL [26]. In another former study, Chinese General Hospital outpatients showed associations between negative illness perceptions and poor mental and physical health status that were similar to those of primary care patients in Western countries [57]. In a cross-sectional study of cancer survivors from Hong Kong greater physical symptom distress and lower levels of optimism were associated with more negative illness perceptions [58].

SOC was found to be an important contributor to both mental and physical health in Chinese General Hospital outpatients [45]. In a more homogenous group of physically ill Chinese breast cancer patients, we have now found that psychological variables play a major role in patients’ QOL.

### Strengths and limitations

Very few studies have assessed the association of physical and psychological variables with QOL in Chinese breast cancer patients. This study population was representative of Chinese breast cancer patients regarding sociodemographic data, cancer stage and treatment [2].

The study also had some limitations: (1) The correlations demonstrated associations, and thus causality could not be inferred. Accordingly, the degree to which patients’ illness-related thoughts and emotions were the consequence of breast cancer and its treatment cannot be determined. (2) We controlled for treatment but not for initial treatment or different pretreatments. (3) It should be noted that the research approach used a Western biopsychosocial model of illness. Therefore, possible culture-specific characteristics may not have been identified, even though there might be cross-cultural similarities in QOL of breast cancer patients.

## Conclusions

The major clinical implication of this study is the broadened view on psychological variables and their associations with physical, mental and social aspects of QOL. In addition to negative affectivity (e.g., depression and anxiety), which is well known, dysfunctional illness perceptions and low SOC in breast cancer patients should also be routinely addressed by Chinese physicians. A simple measure of the cognitive, affective, and behavioral aspects associated with bothersome somatic symptoms may be a helpful tool for screening [59]. Future studies should explore the possible cultural variations in psychological factors, e.g., illness perception and illness attribution in Chinese breast cancer patients, using qualitative methods.

## Abbreviations

BIPQ: The brief illness perception questionnaire
EORTC QLQ-C30: The European Organization for Research and Treatment of Cancer Core Quality of Life Questionnaire-30
GAD-7: General Anxiety Disorder −7
PHQ-15: Patient Health Questionnaire −15
PHQ-9: Patient Health Questionnaire −9
PLA: People’s Liberation Army
QOL: Quality of life
SD: Standard deviation
SOC-9: Sense of coherence
SPSS: is a statistic programm
TCM: Traditional Chinese Medicine
W-7: Whiteley-7

## References

[1] Ferlay J, Soerjomataram I, Dikshit R, Eser S, Mathers C, Rebelo M (2015). Cancer incidence and mortality worldwide: sources, methods and major patterns in GLOBOCAN 2012. Int J Cancer.

[2] Fang QY, Wu Q, Zhang XL, Ma XL (2012). Analysis of the prevalence of the breast cancer. Chi J Soc Med.

[3] Report CCRA (2013). In: national center for cancer registry, disease prevention and control bureau, MOH, editors.

[4] Fan L, Strasser-Weippl K, Li JJ, St Louis J, Finkelstein DM, KD Y, et al. Breast cancer in China. Lancet Oncol. 2014; doi:10.1016/S1470-2045(13)70567-9.

[5] Zeng H, Zheng R, Zhang S, Zou X, Chen W (2014). Female breast cancer statistics of 2010 in China: estimates based on data from 145 population-based cancer registries. J Thorac Dis.

[6] American Society of Clinical Oncology (1996). Outcomes of cancer treatment for technology assessment and cancer treatment guidelines. J Clin Oncol.

[7] Ferrell BR, Grant M, Funk B, Otis-Green S, Garcia N (1997). Quality of life in breast cancer. Part I: physical and social wellbeing. Cancer Nurs.

[8] Ferrell BR, Grant M, Funk B, Otis-Green S, Garcia N (1998). Quality of life in breast cancer. Part II: psychological and spiritual well-being. Cancer Nurs.

[9] Kornblith AB, Powell M, Regan MM, Bennett S, Krasner C, Moy B (2007). Long-term psychosocial adjustment of older vs younger survivors of breast and endometrial cancer. Psychooncology.

[10] Montazeri A. Health-related quality of life in breast cancer patients: A bibliographic review of the literature from 1974 to 2007. J Exp Clin Cancer Res. 2008;doi: 10.1186/1756-9966-27-32. 10.1186/1756-9966-27-32 PMC2543010 18759983

[11] Høyer M, Johansson B, Nordin K, Bergkvist L, Ahlgren J, Lidin-Lindqvist A (2011). Health-related quality of life among women with breast cancer - a population-based study. Acta Oncol (Madr).

[12] Lehto US, Ojanen M, Kellokumpu-Lehtinen P (2005). Predictors of quality of life in newly diagnosed melanoma and breast cancer patients. Ann Oncol.

[13] Mehnert A, Brähler E, Faller H, Härter M, Keller M, Schulz H (2014). Four-week prevalence of mental disorders in patients with cancer across major tumor entities. J Clin Oncol.

[14] Lam WW, Bonanno GA, Mancini AD, Ho S, Chan M, Hung WK (2010). Trajectories of psychological distress among Chinese women diagnosed with breast cancer. Psychooncology.

[15] Wang F, Liu J, Liu L, Wang F, Ma Z, Gao D (2014). The status and correlates of depression and anxiety among breast-cancer survivors in eastern China: a population-based, cross-sectional case-control study. BMC Public Health.

[16] Yan B, Yang LM, Hao LP, Yang C, Quan L, Wang LH, et al. Determinants of Quality of Life for Breast Cancer Patients in Shanghai, China. PLoS ONE. 2016;doi: 10.1371/journal.pone.0153714. 10.1371/journal.pone.0153714 PMC4833339 27082440

[17] Broadbent E, Petrie KJ, Main J, Weinman J (2006). The brief illness perception questionnaire. J Psychosom Res.

[18] Antonovsky A (1979). Health, stress and coping.

[19] Eriksson M, Lindstrom B (2006). Antonovsky’s sense of coherence scale and the relation with health: a systematic review. J Epidemiol Community Health.

[20] Kaptein AA, Schoones JW, Fischer MJ, Thong MSY, Kroep JR, van der Hoeven KJM (2015). Illness perceptions in women with breast cancer—a systematic literature review. Curr Breast Cancer Rep.

[21] Lindner OC, McCabe MG, Radford J, Mayes A, Talmi D, Wearden, A. Quality of life following cancer treatment: impact of illness perceptions, distress, fatigue, and cognitive failures. The European Health Psychologist. 2015;17 Suppl, 629.

[22] Chaturvedi SK, Maguire GP, Somashekar BS (2006). Somatization in cancer. Int Rev Psychiatry.

[23] Gray NM, Hall SJ, Browne S, Johnston M, Lee AJ, Macleod U (2014). Predictors of anxiety and depression in people with colorectal cancer. Support Care Cancer.

[24] Mickeviciene A, Vanagas G, Jievaltas M, Ulys A (2013). Does illness perception explain quality of life of patients with prostate cancer?. Medicina (Kaunas).

[25] Antonovsky A (1987). Unraveling the mystery of health - how people manage stress and stay well.

[26] Zhang Y, Fritzsche K, Leonhart R, Zhao X, Zhang L, Wei J (2014). Dysfunctional illness perception and illness behaviour associated with high somatic symptom severity and low quality of life in general hospital outpatients in China. J Psychosom Res.

[27] Wu H, Zhao X, Fritzsche K, Leonhart R, Schäfert R, Sun X (2015). Quality of doctor-patient relationship in patients with high somatic symptom severity in China. Complement Ther Med.

[28] Leonhart R, Tang L, Pang Y, Li J, Song L, Fischer I (2017). Physical and psychological correlates of high somatic symptom severity in Chinese breast cancer patients. Psychooncology.

[29] Aaronson NK, Ahmedzai S, Bergman B, Bullinger M, Cull A, Duez NJ (1993). The European Organization for Research and Treatment of cancer QLQ-C30: a quality-of-life instrument for use in international clinical trials in oncology. J Natl Cancer Inst.

[30] Fayers PM, Aaronson NK, Bjordal K, Groenvold M, Curran D, Bottomley A. On behalf of the EORTC quality of life group. The EORTC QLQ-C30 scoring manual. 3rd ed. Brussels: European organisation for research and treatment of. Cancer. 2001; http://www.eortc.be/qol/files/SCManualQLQ-C30.pdf

[31] Zhao H, Kanda K (2004). Testing psychometric properties of the standard Chinese version of the European Organization for Research and Treatment of cancer quality of life Core questionnaire 30 (EORTC QLQ-C30). J Epidemiol.

[32] Wan C, Meng Q, Yang Z, Tu X, Feng C, Tang X, Zhang C (2008). Validation of the simplified Chinese version of EORTC QLQ-C30 from the measurements of five types of inpatients with cancer. Ann Oncol.

[33] Cheng J, Liu B, Zhang X, Zhang Y, Lin W, Wang R (2011). The validation of the standard Chinese version of the European Organization for Research and Treatment of cancer quality of life Core questionnaire 30 (EORTC QLQ-C30) in pre-operative patients with brain tumor in China. BMC Med Res Methodol.

[34] Kroenke K, Spitzer RL, Williams JBW (2002). The PHQ-15: validity of a new measure for evaluating the severity of somatic symptoms. Psychosom Med.

[35] Lee S, Ma YL, Tsang A (2011). Psychometric properties of the Chinese 15-item patient health questionnaire in the general population of Hong Kong. J Psychosom Res.

[36] Qian J, Ren Z, Yu D, He X, Li C (2014). Application of patient health questionnaire somatic symptom scale in the general hospital outpatients. Chin Mental Health J.

[37] Kroenke K, Spitzer RL (2002). The PHQ-9: a new depression diagnostic and severity measure. Psychiatr Ann.

[38] Xiong N, Fritzsche K, Wei J, Hong X, Leonhart R, Zhao X (2015). Validation of patient health questionnaire (PHQ) for major depression in Chinese outpatients with multiple somatic symptoms: a multicenter cross-sectional study. J Affect Disord.

[39] Spitzer RL, Kroenke K, Williams JBW, Löwe B (2006). A brief measure for assessing generalized anxiety disorder: the GAD-7. JAMA Intern Med.

[40] He X, Li C, Qian J, Cui H, Reliability WW (2010). Validity of a generalized anxiety scale in general hospital outpatients. Shanghai Arch Psychiatry.

[41] Fink P, Ewald H, Jensen J, Sørensen L, Engberg M, Holm M, Munk-Jørgensen P (1999). Screening for somatization and hypochondriasis in primary care and neurological in-patients: a seven-item scale for hypochondriasis and somatization. J Psychosom Res.

[42] Lee S, Ng KL, Ma YL, Tsang A, Kwok KPA (2011). General population study of the Wihteley-7 index in Hong Kong. J Psychosom Res.

[43] Lin YP, Chiu KM, Wang TJ (2011). Reliability and validity of the Chinese version of the brief illness perception questionnaire for patients with coronary heart disease. J oriental institute of. Technology.

[44] Eriksson M, Lindstrom B (2005). Validity of Antonovsky’s sense of coherence scale: a systematic review. J Epidemiol Community Health.

[45] Li W, Leonhart R, Schaefert R, Zhao X, Zhang L, Wei J (2015). Sense of coherence contributes to physical and mental health in general hospital patients in China. Psychol Health Med..

[46] Hair JF, Black CB, Babin BJ, Anderson RE (2010). Multivariate data analysis.

[47] Fan L, Zheng Y, KD Y, Liu GY, Wu J, JS L (2009). Breast cancer in a transitional society over 18 years: trends and present status in shanghai, China. Breast Cancer Res Treat.

[48] Li T, Mello-Thoms C, Brennan PC (2016). Descriptive epidemiology of breast cancer in China: incidence, mortality, survival and prevalence. Breast Cancer Res Treat.

[49] Peng ZX, Wei J, XS L, Zheng H, Zhong XR, Gao WG (2016). Treatment and survival patterns of Chinese patients diagnosed with breast cancer between 2005 and 2009 in Southwest China an observational, population-based cohort study. Long. W. Medicine.

[50] Zheng Y, Chun-Xiao WU, Zhang ML (2013). The epidemic and characteristics of female breast cancer in China. China. Oncology.

[51] Li X, Huang ML, Hu J, Zeng YCH (2016). Analysis of work related cognitive symptoms of breast cancer survivors and its related factors. Chinese general. Pract Nurs.

[52] Jim HSL, Phillips KM, Chait S, Faul LA, Popa MA, Lee YH (2012). Meta-analysis of cognitive functioning in breast cancer survivors previously treated with standard-dose chemotherapy. J Clin Oncol.

[53] Huang RL, Huang Y, Tao P, Li H, Wang Q, Li H, et al. Evaluation of the quality of life in patients with breast cancer at different TNM stages after standardized treatment.Zhonghua Zhong Liu Za Zhi. 2013;35(1):71–7. doi: 10.3760/cma.j.issn.0253-3766.2013.01.016. 10.3760/cma.j.issn.0253-3766.2013.01.016 23648306

[54] Yan B, Yang LM, Hao LP, Yang C, Quan L, Wang LH (2016). Determinants of quality of life for breast cancer patients in shanghai, China. Lafrenie RM. PLoS One.

[55] So WK, Marsh G, Ling WM, Leung FY, Lo JC, Yeung M (2010). Anxiety, depression and quality of life among Chinese breast cancer patients during adjuvant therapy. Eur J Oncol Nurs.

[56] Hyphantis T, Tomenson B, Paika V, Almyroudi A, Pappa C, Tsifetaki N (2009). Somatization is associated with physical health-related quality of life independent of anxiety and depression in cancer, glaucoma and rheumatological disorders. Qual Life Res.

[57] Wu H, Zhao X, Fritzsche K, Salm F, Leonhart R, Wei J (2014). Negative illness perceptions associated with low mental and physical health status in general hospital outpatients in China. Psychol Health Med.

[58] Zhang N, Fielding R, Soong I, Chan KK, Tsang J, Lee V (2016). Illness perceptions among cancer survivors. Support Care Cancer.

[59] Toussaint A, Murray AM, Voigt K, Herzog A, Gierk B, Kroenke K (2016). Development and validation of the somatic symptom disorder-B criteria scale (SSD-12). Psychosom Med.

